# A case study on the early stage of *Pinus nigra* invasion and its impact on species composition and pattern in Pannonic sand grassland

**DOI:** 10.1038/s41598-024-55811-1

**Published:** 2024-03-01

**Authors:** László Bakacsy, Ágnes Szepesi

**Affiliations:** Department of Plant Biology, Institute of Biology, Faculty of Science and Informatics, University of Szeged, Szeged, 6726 Hungary

**Keywords:** Ecology, Biodiversity, Community ecology, Conservation biology, Grassland ecology, Invasive species

## Abstract

Alien woody species are successful invaders, frequently used for afforestation in regions like semi-arid lands. Shrubs and trees create important microhabitats in arid areas. Understorey vegetation in these habitats has unique species composition and coexistence. However, the impact of solitary woody species on understorey vegetation is less understood. This study evaluated the effect of native (*Juniperus communis*) and invasive solitary conifers (*Pinus nigra*) on surrounding vegetation, where individuals were relatively isolated (referred to as solitary conifers). The field study conducted in Pannonic dry sand grassland in 2018 recorded plant and lichen species presence around six selected solitary conifers. Composition and pattern of understorey vegetation were assessed using 26 m belt transects with 520 units of 5 cm × 5 cm contiguous microquadrats. Compositional diversity (CD) and the number of realized species combinations (NRC) were calculated from the circular transects. Results showed native conifer *J. communis* created more complex, organized microhabitats compared to alien *P. nigra*. CD and NRC values were significantly higher under native conifers than invasive ones (*p* = 0.045 and *p* = 0.026, respectively). Native species also had more species with a homogeneous pattern than the alien species. Alien conifers negatively affected understorey vegetation composition and pattern: some species exhibited significant gaps and clusters of occurrences along the transects under *P. nigra*. Based on our study, the removal of invasive woody species is necessary to sustain habitat diversity.

## Introduction

Plant invasion is a major threat to biodiversity worldwide^1^, with invasive woody species being considered the most problematic invaders in many areas^2,3^. Despite this, alien woody species are often used to create forests for industrial purposes, reforestation, combating desertification or preventing soil erosion^4,5^. However, these species frequently escape from cultivation areas and invade natural and semi-natural habitats, leading to a decline in native plant diversity in these areas^4,6^. They can transform ecosystems and their functioning, strongly compete with native species for space, resources and light, and ultimately have a drastic effect on ecosystem services^2,5,7^. These altered ecosystems can act as starting points for further biological invasions^8–10^.

Native woody species have been reported to create important microhabitats in (the open area of) semi-arid lands^11,12^. Large shrubs or trees act as nurse plants for herbaceous species in arid and semi-arid environment^13^. The soil characteristics e.g. organic and inorganic matter, moisture, temperature and light intensity under and near shrub or tree canopies often significantly differ from the open space of these habitats without trees^14–20^. These microhabitats created by native trees are important because they can alter the plant production and establishing a unique species composition and coexistence^16,21^. Furthermore, every woody species has different effects on the understorey plants as they modify the understorey environments^16,17,22–24^. Some studies have also shown that soil seed bank properties also depend on the species of woody plants^25–27^. However, very few studies have been conducted about the diversity characteristics of the microhabitat under the invasive woody species in semi-arid environments compared to native ones^17,28,29^.

Vegetations of arid and semi-arid lands are resistant to several environmental stress factors, however, they are threatened by plant invasions^30^. The Pannonic dry sand grasslands or open sand grasslands (Natura 2000 code: 6260) are endemic habitats severely sensitive to plant invasion. These are habitats characterized by the dominance of medium to tall perennial grasses or dwarf shrubs that form tufts, with a patchy ground cover. They are associated with communities of annual plants that have developed on either shifting or stable sands, including river sands and ancient dune systems, within the distribution range of the Pannonian steppes^7,31,32^. These open sand grasslands are important unique arid ecosystems of the European Union as are habitats of several protected species such as *Alcanna tinctoria* (L.) Tausch, *Astragalus varius* S.G.Gmel., *Corispermum canescens* Kit., *Corispermum nitidum* Kit., *Festuca wagneri* Thaisz et Flatt, *Sedum urvillei* subsp. *hillebrandtii* (Fenzl) D.A. Webb^32–34^. Common juniper (*Juniperus communis* L.) is the only native conifer species in the Pannonic sand grasslands. They are located in long distances from each other providing scattered occurrence in the open sand grassland matrix. Certain terricolous cryptogam species (mosses and lichens) grow almost exclusively in the shade of junipers^15,35^. However, several drought and heat tolerant invasive woody species are used for wood industry and for the sand stabilization near these habitats^7,18,36^. One of the most frequently used invasive conifer woody species for the afforestation of these areas is *Pinus nigra* J.F. Arnold (black pine or Austrian pine)^37–39^. Because of these forestation practice, black pine is able to spread its seeds by wind to the protected areas outside from the cultivation area^36–38,40^. Thus, this species can easily escape from these planted forests to the nearby sandy grasslands, where their presence is highly undesirable from a nature conservation point of view.

In general, the invasion of pines generally progresses in two waves further into native vegetation: the first begins with the dispersal of seeds from plantation individuals, which creating scattered individuals (wilding plants) on the outer edge of natural vegetation. The second wave of invasion occurs when these scattered individuals on the periphery become sexually mature, and their seeds spread even deeper into the natural vegetation. However, the effect of the solitary trees of the first stage on the vegetation has been thought to be low competition between species^41^. The *Pinus* genus can be a good model for researches of the invasive plant ecology^42^. Understanding the early stage of pine (and other invasive plants as well) invasion processes would be essential from a nature conservation point of view. However these are often overlooked in recent studies due to the challenging to study methodologically. Therefore we evaluate the effects of solitary conifers. Comparing the differences in the understory beta diversity of the native *J. communis* and the alien *P. nigra* in a semi-arid habitat, the Pannonian open sandy grassland. Assuming, these woody species affect on the understorey vegetation in arid and semi-arid ecosystems, we compared the structure and composition of the understorey vegetation of the co-occurring native to alien conifers with information theory method. The aim of the research is to better understand the relationship between woody species and understorey vegetation and the initial stage of invasion processes.

## Material and methods

### Study area

**Figure 1:**
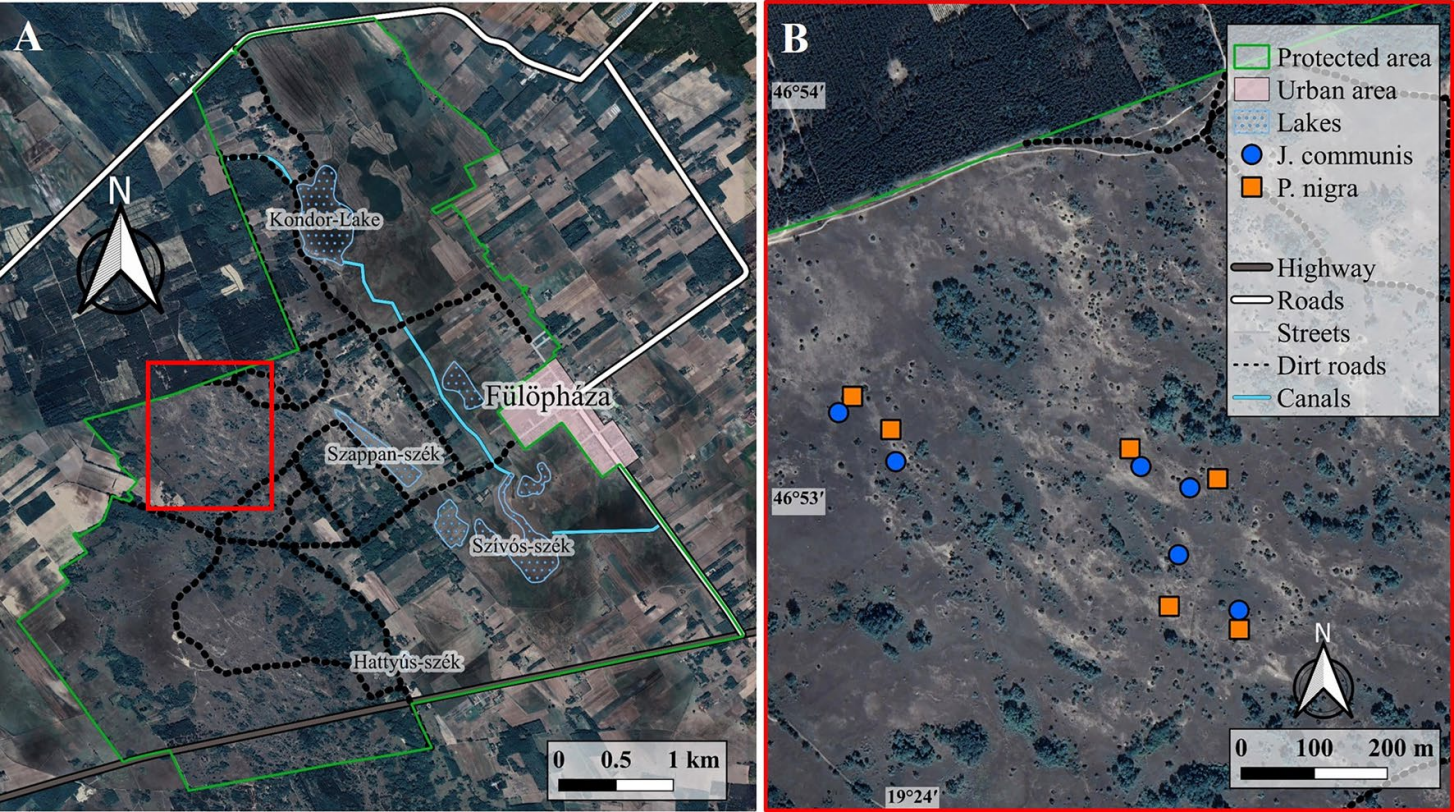
The study area. (**A**) The special protected Fülöpháza Sand Dunes, as a part of Kiskunság National Park in Central Hungary. (**B**) Location of the randomly selected native and alien conifers.

The study was performed in a strictly protected UNESCO biosphere reserve core area, near Fülöpháza in the Kiskunság National Park, Central Hungary (Fig. 1). The map of the study site was edited by QGIS software (version 2.18.24)^43^. The GPS coordinates of the centroid of study area are N46° 52.92ʹ, E0 19° 23.94ʹ. The study area belongs to the Pannonic sand steppes (Natura 2000 category: 6260) of special importance for the European Union Habitat Directive (92/43/CEE)^32,44^. Climate conditions are semiarid temperate with continental and sub-Mediterranean features^32,44^. The mean annual precipitation is 520–550 mm^44^ and the groundwater level is deeper than 2 m^45,46^. The mean annual temperature is 10 °C^44,47,48^. The soil profile is weak, developed on calcareous sand^47–49^. The study area was used as grazing and land for military trainings before protection and abandonment since 1974^50,51^. The vast majority of this semiarid habitat belongs to the open perennial grassland, *Festucetum vaginatae* (its characteristic species are *Festuca vaginata* Waldst. et Kit., *Fumana procumbens* (Dun.) Gren. et Godr., *Stipa borysthenica* Klokov and *Stipa capillata* L., *Euphorbia seguieriana* Neck., *Koeleria glauca* (Spreng.) DC. and *Alyssum tortuosum* Willd.) with solitary junipers (*J. communis*) and rarely covered with forest-steppe vegetation mosaic where the *Populus alba* L. and *P.* × *canescens* Sm. forms small forests of the association *Junipero-Populetum*^15,32,52^. This area is surrounded by agricultural areas, pastures and non-native tree plantations of *Pinus nigra* (Fig. 1). These dry, nutrient-poor, calcareous sand habitats are home to a large number of rare, endangered or endemic species both vascular plants (*Colchicum arenarium* Waldst. et Kit.*, Dianthus diutinus* Kit.*, Dianthus serotinus* Waldst. et Kit.*, Ephedra distachya* L.*, Iris arenaria* Waldst. et Kit.*, Onosma arenaria* Waldst. et Kit.) and lichens (*Cladonia convoluta* (Lam.) Anders.*, Cladonia furcata* (Huds.) Schrad.*, Cladonia magyarica* Vain. ex Gyeln.*, Cladonia rangiformis* Hoffm.*, Toninia physaroides* (Opiz.) Zahlbr.)^32–34^.

### Experimental design

Canopy heights and diameters of the two conifer species individuals also were measured in order to avoid the differences in species coexistence of the surrounding vegetation caused by the different canopy size of the two conifer.

Native species diversity is a commonly used indicator of the impacts of invasive species^53,54^. Although, this is a rather rough means of estimation of the compositional richness of vegetation because only the number of elements of a complex pattern is enlisted without studying the relations between the elements and scales^55^. Therefore, measurements based only the changes in species number are not enough, to detect alterations of diversity more sensitive measures are required. Consequently, the microcoenological structural analysis can be a promising candidate to examine a changes of community^56,57^ and the effect of invasions, respectively^58–60^. Microcoenological structural analysis is a research method and function—developed by Pál Juhász-Nagy—that deals with the internal diversity, order, and coexistence modes and conditions of plant communities. This method and functions are capable of quantitatively describing changes in organizational state, spatial and temporal transitions of vegetation, as well as dynamic and functional aspects^61,62^. Although the method may require significant labor for the unit of area, in recent times, field sampling and data processing methods have been developed that can reduce the workload^61^. Microcoenological analyses are more sensitive to finer spatial and temporal changes than classical phytosociological methods, making them more effective in examining dynamic and evolutionary aspects^61,63^.

The comparative microcoenological structural study was conducted in July, 2018. Six native solitary common junipers (*J. communis*) and six alien solitary black pines (*P. nigra*) were selected randomly to compare their surrounding vegetations in the open sand grassland (Fig. 1). The GPS coordinates of the solitary conifers are in the Supplement (Table S1). The selection criteria of native and alien conifers were appropriate same size of the canopy diameter and height, as it can be seen in the Table 1.

**Table 1:** The studied parameters of the two conifer species. Mean (± SE) of heights and crown diameters for the two conifer individuals. Two-tailed t test, n = 6.

Conifer species	Height (m)	Crown diameter (m)
*Juniperus communis* (native)	4.33 ± 0.33	2.66 ± 0.06
*Pinus nigra* (alien)	4.83 ± 0.42	2.61 ± 0.07
Significance (*p*)	0.3741 (ns)	0.6288 (ns)

**Figure 2:**
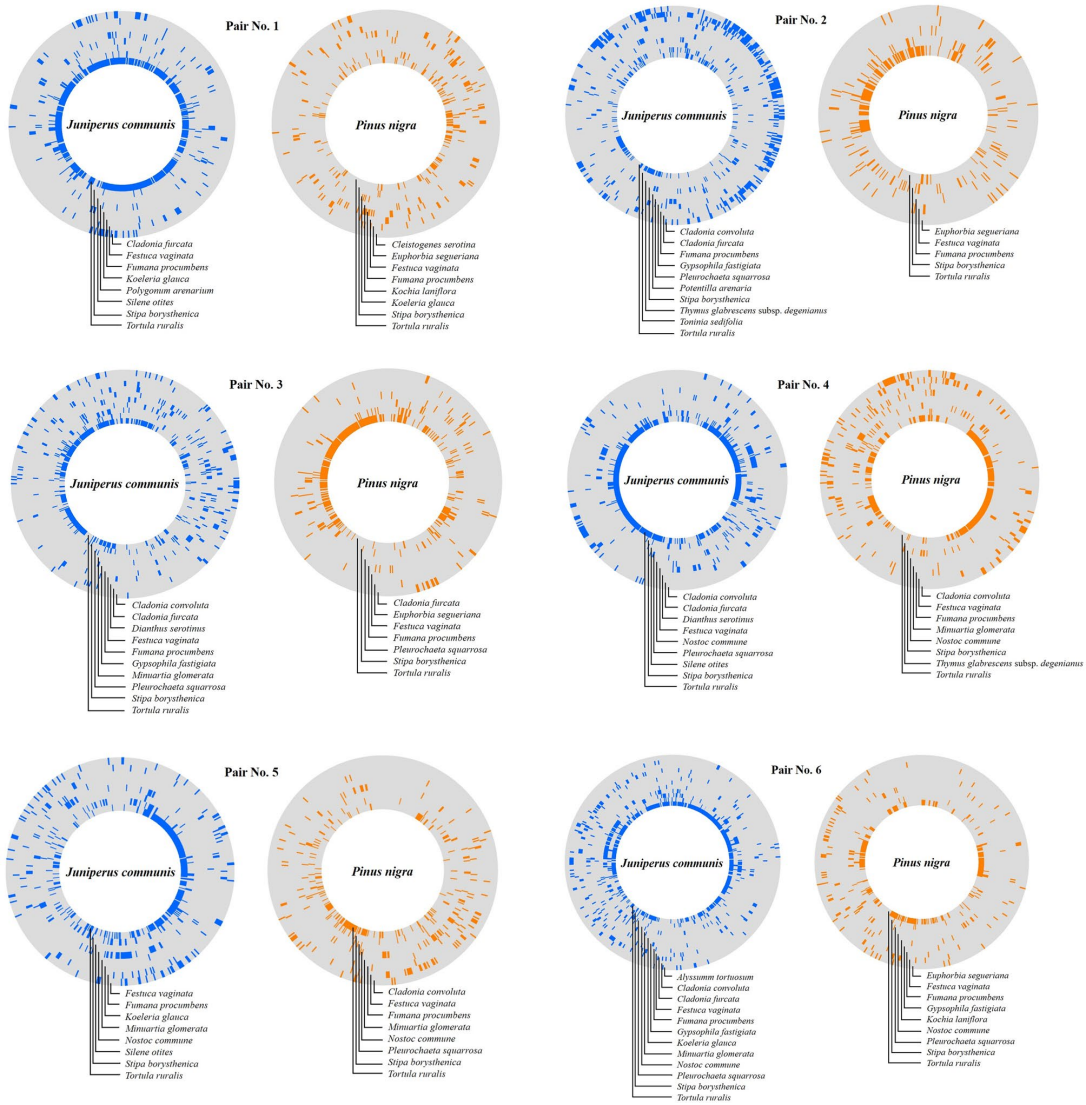
The species presence patterns along 26 m × 5 cm circular transects. The surrounding vegetation of six pair of native conifer (*Juniperus communis*) and six alien conifer (*Pinus nigra*) were selected for illustration. To avoid bias, the number of species with frequency higher than 2% is identical in the two transect. Species are plotted from the outside inward in the circular transects in alphabetical order. The colored lines show the presence of the individual species, the gray gaps show their absence.

Standardized microcoenological sampling protocols were used to detect beta diversity differences between the surrounding vegetations of the two conifers. Therefore, we used a 26 m long belt transect, which was laid around each selected conifer canopy and closed on itself. Furthermore, the transect was placed at a distance of 2 m (± 0.5 m) to the edge of the canopy. Every belt transect consists of 520 units of 5 cm × 5 cm continuous microquadrats^64^. The presence of every plant and lichen species was recorded in every 5 cm × 5 cm microquadrat along each transect^61^ (Fig. 2). The nomenclature of the vascular plant species followed by Király^34^ while in the case of moss and lichen species, it was by Kecskés et al.^65^.

### Data analyses

Microcoenological data were used to the calculation of species compositions, abundance and the procession of a kind of information theory functions (herafter JNP-functions)^66,67^. We used two of these functions in the study. These functions inform us about the composition and structure of a community. The differences between vegetations in these functions are sensitive indicators of beta diversity^44,64^. The compositional diversity (CD) describes the Shannon diversity of species combinations. The number of realized species combination (NRC) display the number of species combinations as a function of spatial resolution. It shows how the frequency and spatial pattern of the species of a vegetation contributes to the formation of community patterns^55–57^.

JNP-functions were calculated by computerised spatial series analyses. For this, the transects data were resampled with different unit sizes, allowing for the estimation of mean species richness at different scales. The functions were calculated across range of spatial scales from 5 cm × 5 cm to 5 cm × 500 cm by merging adjacent microquadrats (merging two, then three, then four… etc.) from the transect data. These results in one CD and one NRC curve for each transect. The curve of one function has two important parameters: maximum value (max. value) and characteristic area (CA, area where the function reaches the max. value). The CA and max. values of the curves were compared to each other: higher max. values indicate more diverse community, as several species are combined in them^61,62^. The CA can be given by the projection of the max. value to the spatial scale. However, in the case of the CA, the smaller area indicates a more diverse community if the given maximum CA presents in a smaller scale^44,63,64,67^.

The two JNP-functions describe the fine-scale structural complexity in a community. Therefore, random references should be used: if the biotic interactions exist, there are significant differences between the field estimates of the two functions and the random expectation^68–70^.

### Statistical analysis

The JNP-information statistic functions, spatial series analyses and random references were calculated by INFOTHEM 3.01 program^69^. The rare species with abundance under 2% were excluded from the analyses those can lead misleading results^71,72^. There are larger effects of an alien species on common species than the rare ones^53,54^. To examine the significance between JNP function values and the given null model, Monte-Carlo method was used to produce large number of random references of samples. 5000 complete randomizations (as random references) and α = 0.01 significance level were applied in each transects^73^.

TBtools program (1.0987671 version) was used to represent the presence of species along the circular transects^74^ (Fig. 2).

The normal distribution of the crown height, diameter, compositional diversity and the number of realized species combinations was examined using the Shapiro–Wilk test (*p* ≤ 0.05). The heights and crown diameters were analysed with parametric two-tailed t test, in order to check whether there are significant differences between the native and invasive conifers. The differences of the two JNP-functions in maximum values and the characteristic areas of the 12 transects were analysed with parametric two-tailed t test. Results were considered significant at *p* ≤ 0.05. The tests and the diagrams were performed in GraphPad Prism version 8.0.1.244 for Windows (GraphPad Software, La Jolla, California, USA).

## Results

This study examined the differences between the beta diversity of the surrounding vegetation of the solitary native and alien conifer species by a comparative multiscale methodology. Two information theory functions were used to determine and compare the beta diversity of the solitary native and alien conifer species surrounding vegetation.

For both species, the average crown height and diameter was over 4 m and 2 m. The crown parameters of *J. communis* and *P. nigra* did not differ significantly (two-tailed t test, heights *p* = 0.374 and crown diameters *p* = 0.628) (Table 1).

A significant difference was observed between the field and complete randomized averages of the two functions values of the transects, regardless of under the natural or invasive species in almost every spatial step (Table S2).

The spatial organization of plant communities below the invasive trees were more heterogeneous in contrast to the native ones. *C. furcata*, *C. convuluta* and *K. glauca* occur more often under the native species and only one species *E. segueriana* occurred with high frequency under the alien species. At the same time, examples of *F. procumbens F. vaginata, S. borysthenica* and *Tortula ruralis* (Hedw.) Gartn. Mey., Schreb. are common equally under both species. It can be also seen in Fig. 2 that the number and the frequency of species are higher under the native species. In addition, species presences show more evenly distributed compared to the vegetation under alien ones: the species form larger denser blocks and gaps under the *P. nigra* vegetations (Fig. 2).

**Figure 3:**
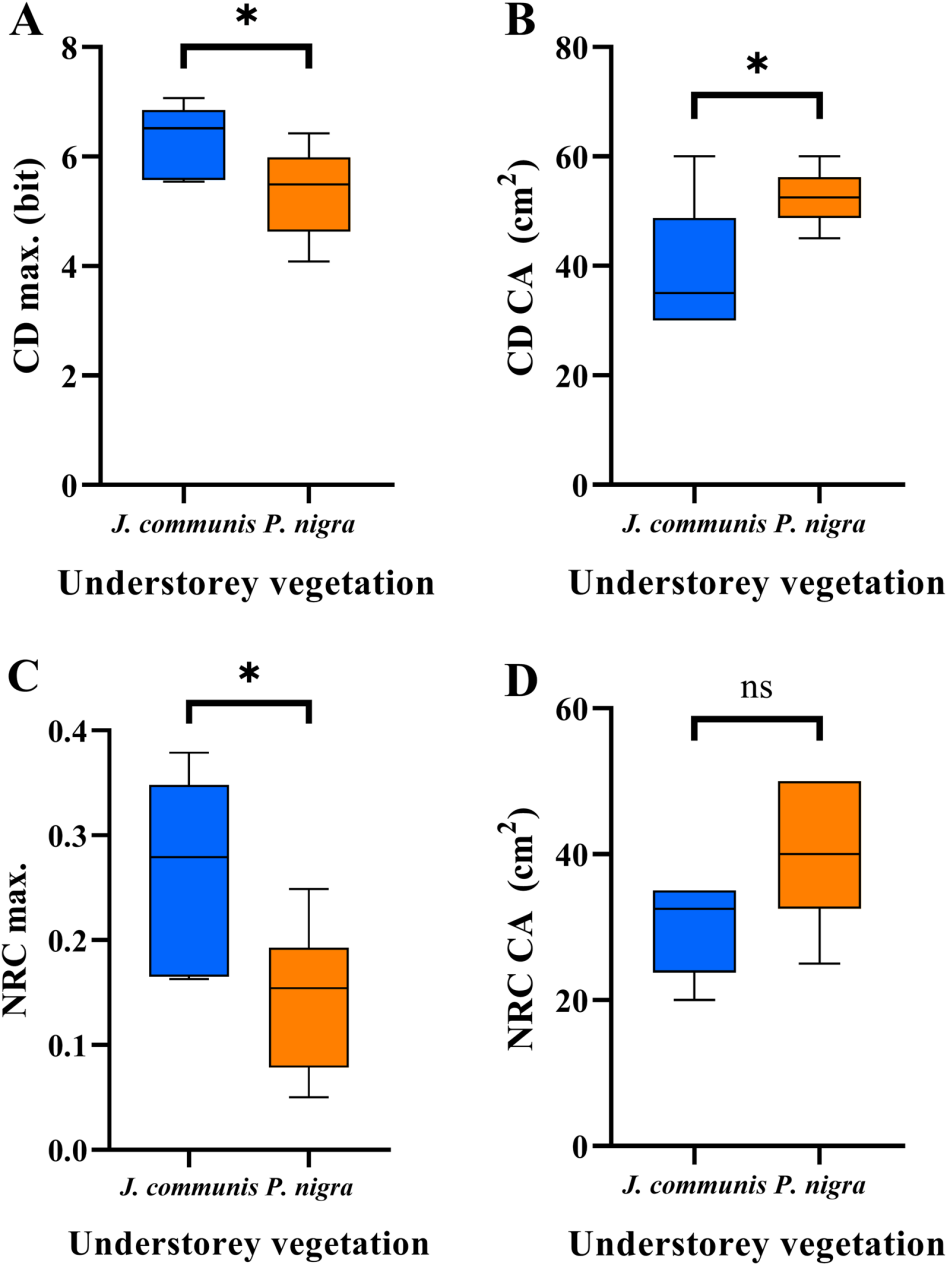
The distribution of the six native and six alien conifer created vegetation in the case of (**A**) The compositional diversity (**C**,**D**) and (**B**) The number of realized species combination (NRC) functions. The maximum values of the two functions and those of the related quadrat sizes were used. To avoid bias, the number of species with frequency higher than 2% is identical in the two transect. Significance level at *p* < 0.05: *, *p* < 0.01: **, *p* < 0.001: *** and insignificant: ns. Two-tailed t test, n = 6.

Based on the two parameters of the used functions (max. values and CA), they showed significant differences between the two microhabitat (native and alien) types (Fig. 3 and Table S3). The max. values of the surrounding vegetations of the *J. communis* (as native) were higher for both functions: the max. values of CD were *p* = 0.045 and in the case of the max. values of NRC was *p* = 0.026.

The max. values of the CD varied between 5.544 and 7.067 bit. The NRC varied between 0.163 and 0.379. In case of the surrounding vegetations on the *P. nigra*, the max. values were lower in both functions. The CD function varied 4.086 to 6.423 bit and the NRC function varied 0.05–0.249 (Fig. 3A,C and Table S3).

There was a significant difference (*p* = 0.028) in the CA of the CD function among the two microhabitats. The CA were in lower scales in the case of native conifer surrounding vegetation, which varied 5 cm × 30 cm–5 cm × 60 cm. While the CA shifted in coarser scale in the *P. nigra* vegetations (5 cm × 45 cm–5 cm × 60 cm) (Fig. 3B and Table S3).

Interestingly, in the CA of the NRC function there was not show a significant different between the two surrounding vegetation. The *J. communis* vegetation had 5 cm × 20 cm–5 cm × 35 cm and *P. nigra* vegetation had 5 cm × 25 cm–5 cm × 50 cm (Fig. 3D and Table S3).

## Discussion

In this study we examined how an alien tree species entering open sandy grasslands modifies the surrounding vegetation by a comparative microcoenology method in the open sand grasslands.

As Naumburg and DeWald^75^ demonstrated, the presence, abundance, and diversity of understory species significantly depended on the size of *Pinus ponderosa* Dougl. ex Laws. Their results suggest that if patches of large trees in the forest structure are scattered, it creates diverse light conditions, resulting in higher diversity of the understory vegetation. Franzese et al.^76^ found that tree height over 4 m significantly influenced the composition of the species under and around the canopy in the case of *P. contorta* Dougl. invasion. That why we selected similar canopy height and diameter of *J. communis* and *P. nigra* in order to avoid artefacts (Table 1). And the average crown height of both species was over 4 m thus, the vegetation may show differences under the two species.

The difference between the randomized average values and the field values indicates that the distribution of the species in a certain vegetation is not random^69^. The same can be seen in this study: in case of both conifer species, biotic interactions form the resulting pattern of both understorey vegetation types between the coexist species (Fig. 2 and Table S2). Fine-scale pattern of species presence demonstrates that species are excluded from alien understorey (Fig. 2), this reduced diversity also (Fig. 3). This was also observed by Szentes et al.^59^ in the case of field study of *Bothriochloa ishaemum* (L.) Keng invasion process and simulated studies^68^.

The vegetations around *P. nigra* have significantly lower max. value of the two functions (CD and NRC) compared to *J. communis* ones (Fig. 3A,C). Moreover, these max. values are shifted in coarser scale (higher CA) in the case of the surrounding vegetation of *P. nigra* (Fig. 3B). These results suggest that the structure of species coexistence is low organized at fine-scales that is, species are not or less able to live together in a small space, close to each other. This suggests that the introduced black pine create a microhabitat that is less suitable for native species. This is consistent with findings from other studies reporting that black pine stands reduced understory richness and diversity. Leege and Murphy^38^ showed that black pines altered the community structure, modified dune habitats and being functionally different from native trees stands (*Pinus banksiana* Lamb. and *Populus deltoids* W.Bartram ex Marshall). Based on the study of Bartha et al.^77^ CD parameters decreased dramatically under *P. nigra* plantation in dolomite grasslands. It seems, that the effects of other arid environments where solitary invasive trees already invaded would be similar to the solitary black pine in Pannonic sand grassland. For instance, the examination of the understorey vegetation around *P. radiata* D.Don individuals clearly shows decreasing of native species cover and richness^28^.

Several studies can provide explanations why common juniper has positive effects on surrounding vegetation while black pine has a negative effect. Both conifer species contain unique secondary metabolic compounds which are emitted by the accumulated litter^78–80^ or endophytic fungi on roots^81,82^. According to Allegrezza et al.^83^ common junipers able to promote the accumulation of organic and inorganic matter, improve soil moisture, mitigate the daily range of soil temperature and thereby, reduce soil evaporation under its canopy in dry grasslands. Interestingly, this facilitation effect of *J. communis* was slight, albeit significantly higher in the disturbed area than in the more stable one^83^. This is supported by Kalapos and Mázsa^15^, who showed that the shade of junipers protects from strong sunlight which could reduce the photochemical efficiency of terricolous cryptogams. Therefore, it seems that the presence of native* J. communis* has a nurse effect on the understorey species. Similar results were obtained by Meiners and Gorchov^84^ when they investigated the relationship between the spatial distribution of native *Juniperus virginiana* L. and tree seedling densities in southwestern Ohio. They showed a significant positive spatial association between tree seedlings and *J. virginiana* in secondary succession. This somewhat contradicts the result of Erfanzadeh et al.^25^, in which the characteristics of the soil seed bank under native *Juniperus sabina* L. were compared with native angiosperms in an arid land of Iran (Baladeh Watershed). According to their results, the density of the soil seed bank was significantly lower under *J. sabina*, but its diversity did not differ. The authors explained the difference in soil seed bank density with the presence of allelochemical substances of *J. sabina*.

The reason behind the decrease in the diversity of native plants may be that invasive Pines alter microhabitat conditions in the invaded ecosystems. Mikulová et al.^85^ found that black pine forest efficiently changed the structural and environmental conditions: microclimate became dryer and warmer, in addition, the amplitude of these parameters were larger. García et al.^86^ reached similar results when investigating the invasion of *Pinus contorta*. The species transforms microenvironmental conditions (i.e. canopy cover, litter accumulation, minimum air temperature and maximum soil temperature) and thereby reduces native plant diversity in the invaded Patagonian ecosystems. In spite of this, the present study did not investigate this conditions, but our results may suggest it. Namely, that several species occurred under the native species, and two lichen species (*C. furcata* and *C. convoluta*) exclusively in the microhabitat formed by *J. communis* (Fig. 2). The negative impacts of *P. nigra* are often related to the accumulated pine needles, which inhibit the development of herbaceous plants^85,87^. Moreover, this can be amplified by the fact that the roots of black pine, including fine roots, can be found in the upper 50 cm of the soil^88^, which effectively reduces soil moister^18^. Similar results have been found by Leege and Murphy^38^ who described, the *P. nigra* sites were significantly drier than of the native *Pinus banksiana* sites in wetpannes. However, the higher frequency of *E. seguriana* under the alien *P. nigra* may also indicate a kind of facilitative relationship. While the occurrence of tussock grasses (*F. vaginata*, *S. borysthenica*) and dwarf shrub *F. procumbens* and moss *T. ruralis* show a neutral relationship, as they occurred with the same frequency under the two species (Fig. 2). This is somewhat confirmed by the results of Langdon et al.^89^, where the young *P. contorta* pines were connected by 25 cm to the native *Baccharis magellanica* (Lam.) Pers. and grasses, which suggests that the spatial distribution of the species included in their study is facilitative interactions may also play a role. However, it has not been investigated what happens when mature pines are present and when the pine crown closes, and what interactions dominate.

The present study also highlights that invasive plant species especially trees were able to alter the understorey vegetation in the early stages of invasion. These degraded microhabitats can provide a starting point for invasions by other non-native species. This is consistent with findings from other studies as non-native conifer forests may be susceptible to invasions due to their lower diversity and smaller coverage of species^9,38,85^. According to a meta-analysis^90^, invasive plants have smaller effects on other neighbouring invasives than natives. In the study of the solitary *Pinus radiata* there were not detectable effects on the understory alien plant cover and richness but the authors acknowledged that exotic plants had a very low abundance^28^. Based on our results, further fine-scale and longer-term studies are necessary to prove this. It remains an open question whether a similar phenomenon can be observed in solitary native and alien angiosperms. And is how the understorey vegetation reacts when the invasive tree species is removed.

## Conclusions

In water limited open habitats the woody species can create unique microhabitats on herb layer communities. This characteristic organization of the understorey vegetation of solitary alien tree species can be examined with comparative fine scale methods. The species composition and diversity of the microhabitat is lower organized under solitary individuals of an invasive tree species found in the open sand grasslands compared to the native ones. The native distribution of *Pinus nigra* is close and its encroachment into the Pannonian region may be due to climate change and the retreat of native species. In longer term, this can have a negative effect on the entire habitat, as it can be the starting point for other invasion processes. Based on the results of this study, it is necessary from a nature conservation point of view to remove the solitary invasive trees to prevent further invasions. In addition, since the solitary trees are derived from the propagule of a nearby *P. nigra* plantation, afforestation methods need to be reconsidered. We recommend that afforestation with non-native tree species be further away from nature conservation areas.

### Supplementary Information

Supplementary Tables.

## Data Availability

This article and its supplementary information files include all data generated or analyzed during this study.
