# Supplementary material for: A case study on the early stage of Pinus nigra invasion and its impact on species composition and pattern in Pannonic sand grassland
Source: Sci Rep. 2024 Mar 1;14:5125. doi: 10.1038/s41598-024-55811-1 (PMC10907687; doi:10.1038/s41598-024-55811-1)
Supplement: Supplementary file 1 — Supplementary Tables. [file 41598_2024_55811_MOESM1_ESM.docx]

**A case study on the early stage of *Pinus nigra* invasion and its impact on species composition and pattern in Pannonic sand grassland**

László Bakacsy* and Ágnes Szepesi

Department of Plant Biology, Institute of Biology, Faculty of Science and Informatics, University of Szeged, Szeged, H-6726, Hungary

*e-mail: [bakacsy@bio.u-szeged.hu](mailto:bakacsy@bio.u-szeged.hu)

Supplementary materials

**Supplementary Table 1.** GPS coordinates of the surrounding vegetations of the solitary conifers which were used in this study. Abbreviation: J1-J6 – surrounding vegetation of the six *J. communis* individuals, P1-P6 – surrounding vegetation of the six *P. nigra* individuals.

| **Conifer species** | **Surrounding vegetation** | **North** | **East** |
| --- | --- | --- | --- |
| *Juniperus communis* (native) | J1 | 46˛53.372' | 019°24.288' |
|  | J2 | 46°53.303' | 019°24.353' |
|  | J3 | 46°53.412' | 019°24.333' |
|  | J4 | 46°53.435' | 019°24.235' |
|  | J5 | 46°53.471' | 019°23.980' |
|  | J6 | 46°53.449' | 019°23.983' |
| *Pinus nigra*  (alien) | P1 | 46°53.319' | 019°24.279' |
|  | P2 | 46°53.310' | 019°24.354' |
|  | P3 | 46°53.409' | 019°24.332' |
|  | P4 | 46°53.434' | 019°24.237' |
|  | P5 | 46°53.471' | 019°23.958' |
|  | P6 | 46°53.446' | 019°23.985' |

**Supplementary Table 2.** The two JNP functions values and significance levels in some spatial series steps of the surrounding vegetations. A) compositional diversity (CD) and B) number of realized species combination (NRF). Abbreviation: J1-J6 – surrounding vegetation of the six *J. communis* individuals, P1-P6 – surrounding vegetation of the six *P. nigra* individuals. JNP functions a – average value of randomization f – field value and *p* – significance level at a given spatial scale (Area). To avoid bias, the number of species with frequency higher than 2% is identical in the two transect.

A)

| J 1 | | | |  | P 1 | | | |
| --- | --- | --- | --- | --- | --- | --- | --- | --- |
| Area (cm^2^) | aCD | fCD | *pCD* |  | Area (cm^2^) | aCD | fCD | *pCD* |
| 5 | 2.713 | 2.696 | *0.127* |  | 5 | 2.664 | 2.668 | *0.395* |
| 50 | 5.788 | 5.114 | *0.0002* |  | 50 | 5.729 | 5.771 | *0.355* |
| 100 | 4.999 | 5.013 | *0.468* |  | 100 | 4.917 | 5.364 | *0.002* |
| 150 | 3.881 | 4.587 | *0.0002* |  | 150 | 4.162 | 4.726 | *0.0008* |
| 200 | 2.945 | 4.097 | *0.0002* |  | 200 | 3.522 | 4.411 | *0.0002* |
| 250 | 2.229 | 3.704 | *0.0002* |  | 250 | 2.953 | 4.044 | *0.0002* |
| 300 | 1.699 | 3.328 | *0.0002* |  | 300 | 2.441 | 3.572 | *0.0002* |
| 350 | 1.308 | 2.902 | *0.0002* |  | 350 | 1.983 | 3.196 | *0.0002* |
| 400 | 1.013 | 2.540 | *0.0002* |  | 400 | 1.581 | 2.791 | *0.0002* |
| 450 | 0.786 | 2.357 | *0.0002* |  | 450 | 1.236 | 2.394 | *0.0032* |
| 500 | 0.608 | 2.254 | *0.0002* |  | 500 | 0.948 | 2.075 | *0.0078* |
| J 2 | | | |  | P 2 | | | |
| Area (cm^2^) | aCD | fCD | *pCD* |  | Area (cm^2^) | aCD | fCD | *pCD* |
| 5 | 4.401 | 4.384 | *0.21* |  | 5 | 2.012 | 2.009 | *0.308* |
| 50 | 5.897 | 6.180 | *0.0028* |  | 50 | 3.564 | 4.084 | *0.0002* |
| 100 | 4.970 | 5.439 | *0.0014* |  | 100 | 3.059 | 3.806 | *0.0002* |
| 150 | 4.072 | 4.650 | *0.0018* |  | 150 | 2.596 | 3.482 | *0.0002* |
| 200 | 3.319 | 4.106 | *0.001* |  | 200 | 2.126 | 2.933 | *0.0002* |
| 250 | 2.706 | 4.076 | *0.0002* |  | 250 | 1.691 | 2.438 | *0.0014* |
| 300 | 2.204 | 3.906 | *0.0002* |  | 300 | 1.317 | 2.049 | *0.0056* |
| 350 | 1.788 | 3.673 | *0.0002* |  | 350 | 1.006 | 1.762 | *0.0102* |
| 400 | 1.439 | 3.533 | *0.0002* |  | 400 | 0.752 | 1.577 | *0.0076* |
| 450 | 1.144 | 3.422 | *0.0002* |  | 450 | 0.553 | 1.507 | *0.003* |
| 500 | 0.898 | 3.277 | *0.0002* |  | 500 | 0.399 | 1.413 | *0.0024* |
| J 3 | | | |  | P 3 | | | |
| Area (cm^2^) | aCD | fCD | *pCD* |  | Area (cm^2^) | aCD | fCD | *pCD* |
| 5 | 4.106 | 4.067 | *0.0454* |  | 5 | 2.515 | 2.488 | *0.0158* |
| 50 | 6.919 | 6.637 | *0.0012* |  | 50 | 4.756 | 4.768 | *0.46* |
| 100 | 5.413 | 5.892 | *0.001* |  | 100 | 4.636 | 4.682 | *0.366* |
| 150 | 3.836 | 5.082 | *0.0002* |  | 150 | 4.039 | 4.286 | *0.071* |
| 200 | 2.625 | 4.725 | *0.0002* |  | 200 | 3.384 | 4.038 | *0.0014* |
| 250 | 1.783 | 4.103 | *0.0002* |  | 250 | 2.778 | 3.788 | *0.0002* |
| 300 | 1.220 | 3.472 | *0.0002* |  | 300 | 2.254 | 3.583 | *0.0002* |
| 350 | 0.849 | 3.001 | *0.0002* |  | 350 | 1.815 | 3.260 | *0.0002* |
| 400 | 0.597 | 2.755 | *0.0002* |  | 400 | 1.453 | 2.863 | *0.0002* |
| 450 | 0.423 | 2.643 | *0.0002* |  | 450 | 1.155 | 2.249 | *0.0018* |
| 500 | 0.301 | 2.409 | *0.0002* |  | 500 | 0.908 | 2.093 | *0.0016* |
| J 4 | | | |  | P 4 | | | |
| Area (cm^2^) | aCD | fCD | *pCD* |  | Area (cm^2^) | aCD | fCD | *pCD* |
| 5 | 3.413 | 3.351 | *0.002* |  | 5 | 3.235 | 3.188 | *0.003* |
| 50 | 5.846 | 5.545 | *0.0044* |  | 50 | 5.376 | 5.244 | *0.0864* |
| 100 | 5.112 | 5.417 | *0.013* |  | 100 | 4.478 | 4.758 | *0.0486* |
| 150 | 4.299 | 5.230 | *0.0002* |  | 150 | 3.665 | 4.326 | *0.0004* |
| 200 | 3.528 | 4.779 | *0.0002* |  | 200 | 3.034 | 3.877 | *0.0002* |
| 250 | 2.834 | 4.470 | *0.0002* |  | 250 | 2.513 | 3.588 | *0.0002* |
| 300 | 2.233 | 3.861 | *0.0002* |  | 300 | 2.066 | 3.183 | *0.0002* |
| 350 | 1.728 | 3.441 | *0.0002* |  | 350 | 1.681 | 2.624 | *0.0012* |
| 400 | 1.312 | 3.147 | *0.0002* |  | 400 | 1.350 | 2.296 | *0.0022* |
| 450 | 0.978 | 2.881 | *0.0002* |  | 450 | 1.070 | 2.067 | *0.0032* |
| 500 | 0.716 | 2.467 | *0.0002* |  | 500 | 0.839 | 1.953 | *0.002* |
| J 5 | | | |  | P 5 | | | |
| Area (cm^2^) | aCD | fCD | *pCD* |  | Area (cm^2^) | aCD | fCD | *pCD* |
| 5 | 3.561 | 3.542 | *0.123* |  | 5 | 2.765 | 2.765 | *0.442* |
| 50 | 5.141 | 5.452 | *0.0022* |  | 50 | 5.788 | 5.739 | *0.305* |
| 100 | 4.141 | 4.797 | *0.0002* |  | 100 | 4.887 | 5.329 | *0.0022* |
| 150 | 3.392 | 4.016 | *0.0004* |  | 150 | 4.065 | 4.633 | *0.0012* |
| 200 | 2.774 | 3.746 | *0.0002* |  | 200 | 3.365 | 3.979 | *0.0016* |
| 250 | 2.238 | 3.406 | *0.0002* |  | 250 | 2.752 | 3.474 | *0.0016* |
| 300 | 1.779 | 3.206 | *0.0002* |  | 300 | 2.212 | 2.939 | *0.0078* |
| 350 | 1.393 | 2.967 | *0.0002* |  | 350 | 1.743 | 2.453 | *0.028* |
| 400 | 1.076 | 2.627 | *0.0002* |  | 400 | 1.345 | 2.225 | *0.0158* |
| 450 | 0.818 | 2.413 | *0.0002* |  | 450 | 1.012 | 2.035 | *0.0092* |
| 500 | 0.614 | 2.210 | *0.0002* |  | 500 | 0.742 | 1.883 | *0.0044* |
| J 6 | | | |  | P 6 | | | |
| Area (cm^2^) | aCD | fCD | *pCD* |  | Area (cm^2^) | aCD | fCD | *pCD* |
| 5 | 4.566 | 4.429 | *0.0002* |  | 5 | 2.704 | 2.701 | *0.404* |
| 50 | 7.122 | 6.922 | *0.014* |  | 50 | 5.871 | 5.816 | *0.284* |
| 100 | 6.126 | 6.333 | *0.0446* |  | 100 | 5.832 | 5.683 | *0.12* |
| 150 | 5.032 | 5.622 | *0.0002* |  | 150 | 5.266 | 5.183 | *0.282* |
| 200 | 3.996 | 4.765 | *0.001* |  | 200 | 4.602 | 4.806 | *0.131* |
| 250 | 3.097 | 4.321 | *0.0002* |  | 250 | 3.924 | 4.368 | *0.0296* |
| 300 | 2.357 | 3.959 | *0.0002* |  | 300 | 3.279 | 4.072 | *0.003* |
| 350 | 1.768 | 3.554 | *0.0002* |  | 350 | 2.689 | 3.840 | *0.0004* |
| 400 | 1.309 | 3.143 | *0.0002* |  | 400 | 2.166 | 3.583 | *0.0002* |
| 450 | 0.957 | 2.849 | *0.0002* |  | 450 | 1.714 | 3.318 | *0.0002* |
| 500 | 0.691 | 2.616 | *0.0002* |  | 500 | 1.331 | 3.005 | *0.0002* |

B)

| J 1 | | | |  | P 1 | | | |
| --- | --- | --- | --- | --- | --- | --- | --- | --- |
| Area (cm^2^) | aNRF | fNRF | *pNRF* |  | Area (cm^2^) | aNRF | fNRF | *pNRF* |
| 5 | 0.066 | 0.056 | *0.0294* |  | 5 | 0.061 | 0.054 | *0.0822* |
| 50 | 0.153 | 0.106 | *0.0002* |  | 50 | 0.168 | 0.164 | *0.393* |
| 100 | 0.101 | 0.090 | *0.107* |  | 100 | 0.097 | 0.116 | *0.0294* |
| 150 | 0.056 | 0.069 | *0.0436* |  | 150 | 0.056 | 0.071 | *0.0314* |
| 200 | 0.033 | 0.054 | *0.0012* |  | 200 | 0.037 | 0.058 | *0.0014* |
| 250 | 0.021 | 0.042 | *0.0004* |  | 250 | 0.027 | 0.051 | *0.0002* |
| 300 | 0.014 | 0.037 | *0.0004* |  | 300 | 0.021 | 0.037 | *0.0004* |
| 350 | 0.011 | 0.029 | *0.0004* |  | 350 | 0.017 | 0.031 | *0.0004* |
| 400 | 0.009 | 0.023 | *0.0006* |  | 400 | 0.014 | 0.023 | *0.015* |
| 450 | 0.007 | 0.016 | *0.003* |  | 450 | 0.011 | 0.019 | *0.03* |
| 500 | 0.006 | 0.017 | *0.0004* |  | 500 | 0.009 | 0.014 | *0.107* |
| J 2 | | | |  | P 2 | | | |
| Area (cm^2^) | aNRF | fNRF | *pNRF* |  | Area (cm^2^) | aNRF | fNRF | *pNRF* |
| 5 | 0.137 | 0.119 | *0.0048* |  | 5 | 0.030 | 0.029 | *0.547* |
| 50 | 0.186 | 0.213 | *0.0218* |  | 50 | 0.044 | 0.049 | *0.182* |
| 100 | 0.100 | 0.140 | *0.0002* |  | 100 | 0.025 | 0.040 | *0.0004* |
| 150 | 0.058 | 0.081 | *0.0024* |  | 150 | 0.018 | 0.035 | *0.0002* |
| 200 | 0.036 | 0.058 | *0.0012* |  | 200 | 0.015 | 0.025 | *0.0002* |
| 250 | 0.025 | 0.047 | *0.0004* |  | 250 | 0.012 | 0.019 | *0.0026* |
| 300 | 0.018 | 0.043 | *0.0002* |  | 300 | 0.010 | 0.015 | *0.037* |
| 350 | 0.014 | 0.038 | *0.0002* |  | 350 | 0.008 | 0.013 | *0.0454* |
| 400 | 0.012 | 0.032 | *0.0002* |  | 400 | 0.007 | 0.009 | *0.324* |
| 450 | 0.010 | 0.030 | *0.0002* |  | 450 | 0.006 | 0.007 | *0.554* |
| 500 | 0.008 | 0.029 | *0.0002* |  | 500 | 0.005 | 0.007 | *0.331* |
| J 3 | | | |  | P 3 | | | |
| Area (cm^2^) | aNRF | fNRF | *pNRF* |  | Area (cm^2^) | aNRF | fNRF | *pNRF* |
| 5 | 0.132 | 0.115 | *0.009* |  | 5 | 0.048 | 0.042 | *0.137* |
| 50 | 0.314 | 0.286 | *0.0318* |  | 50 | 0.087 | 0.088 | *0.486* |
| 100 | 0.139 | 0.172 | *0.0054* |  | 100 | 0.073 | 0.070 | *0.384* |
| 150 | 0.062 | 0.104 | *0.0002* |  | 150 | 0.052 | 0.059 | *0.131* |
| 200 | 0.032 | 0.079 | *0.0002* |  | 200 | 0.037 | 0.044 | *0.124* |
| 250 | 0.019 | 0.062 | *0.0002* |  | 250 | 0.026 | 0.040 | *0.0048* |
| 300 | 0.012 | 0.039 | *0.0002* |  | 300 | 0.020 | 0.041 | *0.0002* |
| 350 | 0.008 | 0.029 | *0.0002* |  | 350 | 0.015 | 0.033 | *0.0004* |
| 400 | 0.006 | 0.023 | *0.0002* |  | 400 | 0.012 | 0.025 | *0.0018* |
| 450 | 0.005 | 0.023 | *0.0002* |  | 450 | 0.010 | 0.016 | *0.0482* |
| 500 | 0.005 | 0.021 | *0.0002* |  | 500 | 0.008 | 0.012 | *0.156* |
| J 4 | | | |  | P 4 | | | |
| Area (cm^2^) | aNRF | fNRF | *pNRF* |  | Area (cm^2^) | aNRF | fNRF | *pNRF* |
| 5 | 0.090 | 0.079 | *0.0318* |  | 5 | 0.074 | 0.067 | *0.132* |
| 50 | 0.178 | 0.143 | *0.0008* |  | 50 | 0.132 | 0.123 | *0.173* |
| 100 | 0.104 | 0.116 | *0.124* |  | 100 | 0.076 | 0.080 | *0.38* |
| 150 | 0.062 | 0.116 | *0.0002* |  | 150 | 0.042 | 0.059 | *0.0116* |
| 200 | 0.041 | 0.077 | *0.0002* |  | 200 | 0.028 | 0.046 | *0.0016* |
| 250 | 0.028 | 0.066 | *0.0002* |  | 250 | 0.021 | 0.036 | *0.0008* |
| 300 | 0.021 | 0.050 | *0.0002* |  | 300 | 0.016 | 0.033 | *0.0002* |
| 350 | 0.016 | 0.035 | *0.0002* |  | 350 | 0.013 | 0.022 | *0.0078* |
| 400 | 0.012 | 0.027 | *0.0006* |  | 400 | 0.011 | 0.016 | *0.0822* |
| 450 | 0.010 | 0.026 | *0.0002* |  | 450 | 0.009 | 0.012 | *0.28* |
| 500 | 0.008 | 0.019 | *0.0016* |  | 500 | 0.008 | 0.012 | *0.107* |
| J 5 | | | |  | P 5 | | | |
| Area (cm^2^) | aNRF | fNRF | *pNRF* |  | Area (cm^2^) | aNRF | fNRF | *pNRF* |
| 5 | 0.084 | 0.069 | *0.004* |  | 5 | 0.065 | 0.063 | *0.491* |
| 50 | 0.122 | 0.147 | *0.0018* |  | 50 | 0.173 | 0.174 | *0.497* |
| 100 | 0.059 | 0.092 | *0.0002* |  | 100 | 0.095 | 0.116 | *0.0208* |
| 150 | 0.034 | 0.055 | *0.0004* |  | 150 | 0.053 | 0.071 | *0.013* |
| 200 | 0.024 | 0.044 | *0.0002* |  | 200 | 0.035 | 0.048 | *0.0192* |
| 250 | 0.018 | 0.030 | *0.0016* |  | 250 | 0.025 | 0.040 | *0.0016* |
| 300 | 0.014 | 0.026 | *0.001* |  | 300 | 0.019 | 0.024 | *0.171* |
| 350 | 0.011 | 0.024 | *0.0004* |  | 350 | 0.015 | 0.018 | *0.324* |
| 400 | 0.009 | 0.018 | *0.0046* |  | 400 | 0.012 | 0.016 | *0.212* |
| 450 | 0.008 | 0.016 | *0.005* |  | 450 | 0.010 | 0.014 | *0.179* |
| 500 | 0.007 | 0.014 | *0.0058* |  | 500 | 0.008 | 0.014 | *0.0492* |
| J 6 | | | |  | P 6 | | | |
| Area (cm^2^) | aNRF | fNRF | *pNRF* |  | Area (cm^2^) | aNRF | fNRF | *pNRF* |
| 5 | 0.171 | 0.140 | *0.0002* |  | 5 | 0.063 | 0.067 | *0.282* |
| 50 | 0.361 | 0.327 | *0.0244* |  | 50 | 0.177 | 0.164 | *0.143* |
| 100 | 0.198 | 0.212 | *0.186* |  | 100 | 0.160 | 0.154 | *0.327* |
| 150 | 0.106 | 0.141 | *0.001* |  | 150 | 0.113 | 0.104 | *0.202* |
| 200 | 0.060 | 0.096 | *0.0002* |  | 200 | 0.077 | 0.083 | *0.272* |
| 250 | 0.037 | 0.079 | *0.0002* |  | 250 | 0.053 | 0.059 | *0.244* |
| 300 | 0.025 | 0.063 | *0.0002* |  | 300 | 0.038 | 0.046 | *0.163* |
| 350 | 0.017 | 0.051 | *0.0002* |  | 350 | 0.028 | 0.044 | *0.006* |
| 400 | 0.013 | 0.034 | *0.0004* |  | 400 | 0.021 | 0.036 | *0.007* |
| 450 | 0.010 | 0.028 | *0.0004* |  | 450 | 0.017 | 0.035 | *0.0012* |
| 500 | 0.008 | 0.024 | *0.0004* |  | 500 | 0.013 | 0.031 | *0.0008* |

**Supplementary Table 3.** The JNP information theory function in the two conifer species surrounding vegetation. J – vegetation around *Juniperus communis*, P – vegetation around *Pinus nigra*. The maximum values (Max. value) and characteristic areas (CA) of the compositional diversity (CD) and number of realized species combinations (NRC) functions were used in the analysis. Significance level at *p* < 0.05: *, *p* < 0.01: **, *p* < 0.001: *** and insignificant: ns. Two-tailed t test, n = 6.

| **JNP- information theory functions** | **Understorey vegetation** | **Max. value** | **CA** | **Understorey vegetation** | **Max. value** | **CA** | **Comparison** | **t-test** (*p*) | |
| --- | --- | --- | --- | --- | --- | --- | --- | --- | --- |
|  | (native) |  |  | (alien) |  |  | (for t-test) |  |  |
| CD | J1 | 6.353 bit | 35 cm × 5 cm | P1 | 4.086 bit | 55 cm × 5 cm |  |  |  |
|  | J2 | 6.683 bit | 45 cm × 5 cm | P2 | 4.808 bit | 60 cm × 5 cm | Max. values | 0.0452 | * |
|  | J3 | 5.579 bit | 60 cm × 5 cm | P3 | 5.244 bit | 50 cm × 5 cm |  |  |  |
|  | J4 | 5.544 bit | 30 cm × 5 cm | P4 | 5.738 bit | 50 cm × 5 cm |  |  |  |
|  | J5 | 7.067 bit | 35 cm × 5 cm | P5 | 5.836 bit | 55 cm × 5 cm | CA | 0.0280 | * |
|  | J6 | 6.78 bit | 30 cm × 5 cm | P6 | 6.423 bit | 45 cm × 5 cm |  |  |  |
| NRC | J1 | 0.236 | 25 cm × 5 cm | P1 | 0.05 | 40 cm × 5 cm |  |  |  |
|  | J2 | 0.322 | 35 cm × 5 cm | P2 | 0.088 | 50 cm × 5 cm | Max. values | 0.0267 | * |
|  | J3 | 0.163 | 35 cm × 5 cm | P3 | 0.137 | 25 cm × 5 cm |  |  |  |
|  | J4 | 0.166 | 30 cm × 5 cm | P4 | 0.174 | 50 cm × 5 cm |  |  |  |
|  | J5 | 0.379 | 35 cm × 5 cm | P5 | 0.171 | 40 cm × 5 cm | CA | 0.0572 | ns |
|  | J6 | 0.338 | 20 cm × 5 cm | P6 | 0.249 | 35 cm × 5 cm |  |  |  |
